# Facile One-Step Electrospinning Process to Prepare AgNPs-Loaded PLA and PLA/PEO Mats with Antibacterial Activity

**DOI:** 10.3390/polym15061470

**Published:** 2023-03-16

**Authors:** Valeria Allizond, Giuliana Banche, Matteo Salvoni, Mery Malandrino, Claudio Cecone, Anna Maria Cuffini, Pierangiola Bracco

**Affiliations:** 1 Department of Public Health and Pediatric Sciences, University of Torino, Via Santena 9, 10126 Turin, Italy; 2 Department of Chemistry, NIS Interdepartmental Centre, University of Torino, Via P. Giuria 7, 10125 Turin, Italy

**Keywords:** nanofibers, silver nanoparticles, electrospinning, antibacterial activity

## Abstract

Nanofibers can play an important role in developing new kinds of medical applications. Poly(lactic acid) (PLA) and PLA/poly(ethylene oxide) (PEO) antibacterial mats containing silver nanoparticles (AgNPs) were prepared by a simple one-step electrospinning method that allows AgNPs to be synthesized simultaneously with the preparation of the electrospinning solution. The electrospun nanofibers were characterized by scanning electron microscopy, transmission electron microscopy and thermogravimetry, while silver release over time was monitored by inductively coupled plasma/optical emission spectroscopy. The antibacterial activity was tested against *Staphylococcus epidermidis* and *Escherichia coli* by colony forming unit (CFU) count on agar after 15, 24 and 48 h of incubation. AgNPs were found to be mainly concentrated in the PLA nanofiber core, and the mats showed steady but slow Ag release in the short term; in contrast, AgNPs were uniformly distributed in the PLA/PEO nanofibers, which released up to 20% of their initial silver content in 12 h. A significant (*p* < 0.05) antimicrobial effect towards both tested bacteria, highlighted by a reduction in the CFU/mL counts, was observed for the nanofibers of PLA and PLA/PEO embedded with AgNPs, with a stronger effect exerted by the latter, confirming the more efficient silver release from these samples. The prepared electrospun mats may have good potential for use in the biomedical field, particularly in wound dressing applications, where a targeted delivery of the antimicrobial agent is highly desirable to avoid infections.

## 1. Introduction

Electrospinning is a highly versatile method to produce continuous fibers, with diameters ranging from a few nanometers to micrometers, from polymer solutions or melts [1,2,3]. Thanks to remarkable features such as the very high surface/volume ratio, the wide compositional and morphological versatility, a tunable porous structure and the ability to conform to a variety of sizes and shapes, since the early 2000s, electrospun fibrous mats have undergone a dizzying development in a broad spectrum of applications, including water and air treatment and purification, catalysis, energy, photonics and electronics, smart materials for various purposes and, most importantly, biomedical applications [4]. The peculiar characteristics of the electrospun matrices have attracted great interest in the field of biomaterials and biomedical devices, because they are often well-suited to meet the complex requirements of advanced applications, such as tissue engineering, drug delivery, wound dressing, enzyme immobilization, etc. [4,5,6]. One of the many attractive possibilities offered by the electrospinning technique is that of incorporating drugs, functional fillers or bioactive molecules inside the fiber core that can subsequently be released over a prolonged period of time [5].

Among the variety of active agents that have been investigated for this purpose, antibacterial agents and antibiotics have played a primary role, since microbial infections are still one of the most important challenges in public health, and the continuous development of multidrug resistant microorganisms makes urgent the need to find new solutions. Many literature studies have reported the successful preparation of electrospun mats based on biocompatible and often biodegradable polymers capable of releasing various antibacterial agents with controllable rates [7,8]. In particular, the well-known anti-microbial properties of silver and its ions have stimulated numerous researches aimed at the preparation of polymeric scaffolds containing silver nanoparticles (AgNPs), capable of implementing an immobilization of the same, together with a sustained antibacterial effect [9,10,11,12]. Recent studies have shown that spherical-shaped AgNPs with sizes below 20 nm demonstrate the highest antibacterial activity [13]; however, handling small-sized AgNPs remains a challenging problem since the particles can be lost during collection. Thus, several researches have shown that the deposition of AgNPs on adequate supports facilitates handling, avoids their aggregation and prevents oxidization, thus boosting the antibacterial activity [14,15]. A wide variety of polymeric matrices have been tested, ranging from natural polymers (cellulose, alginate, chitosan) [16,17,18], to biodegradable [poly(lactic-co-glycolic acid (PLGA), poly(lactic acid) (PLA), polycaprolactone (PCL)] [11,19,20,21,22], to conventional, synthetic polymers [23,24]. Additionally, with regard to the methodology for preparing the silver nanoparticles and their introduction into the fibers, various routes have been explored, ranging from the electrospinning of nanoparticle suspensions in polymer solutions [20,25] to the use of precursors, such as silver salts, and their subsequent physical, chemical or biological reduction [26,27,28,29]. However, each of these methods has some disadvantages: suspensions of AgNPs are generally unstable and NPs tend to agglomerate, therefore capping agents must be used; chemical methods are often complex and time consuming, as they require multiple processing steps; and biological methods, although promising, require careful control of the reaction conditions (i.e., temperature, pH, amount of reducing and stabilizing factors) [30].

In the present work, we aimed to implement a simple method to produce electrospun antibacterial mats based on PLA or blends of PLA and poly(ethylene oxide) (PEO) loaded with AgNPs. We therefore developed a one-step preparation process, carrying out the reduction of AgNO_3_ simultaneously with the preparation of the PLA solution for electrospinning; once the feasibility of the process and the antibacterial activity of the product were verified, we successfully explored the use of PLA/PEO blends to improve short-term silver release. Overall, our results suggest that the prepared electrospun mats may have good potential for use in the biomedical field, particularly in wound dressing applications.

## 2. Materials and Methods

### 2.1. Materials and Processing

PLA with an average molecular weight (M_w_) of 200,000 g/mol was supplied by NatureWorks Co. (Plymouth, MN, USA). PEO with an average molecular weight (M_w_) of 100,000 g/mol, AgNO_3_ (99+%, ACS grade), acetonitrile (≥99.9%), dichloromethane (≥99.8%) and dimethylformamide (≥99.8%) were purchased from Sigma Aldrich—Merck (Merk KGaA, Darmstadt, Germany) and used without further purification.

The electrospinning apparatus consisted of a 3 mL plastic syringe coupled with a volumetric pump and a power supply needed to generate a potential between the syringe’s gauge 21 nozzle (electrode) and the collector (ground). The deposition was carried out at room temperature by setting a working distance of 18 cm, 25 kV field strength and 0.5 mL/h flow, unless otherwise specified. An aluminum plate was used as the collector.

### 2.2. Fiber Characterization

UV-Vis spectrometry was used to monitor the synthesis of AgNPs. A PerkinElmer Lambda 25 UV/Vis spectrometer (PerkinElmer, Waltham, MA, USA) was used in the wavelength range of 350–650 nm at room temperature.

The fiber morphology of electrospun mats was examined by scanning electron microscopy (SEM). The images were acquired with a Leica Stereoscan 410 (Oxford Instruments, Abingdon-on-Thames, UK), using secondary electrons and 15 kV of accelerating voltage, after the sputter-coating of specimens with a gold nanolayer. The average diameter of the nanofibers was determined using ImageJ software, from at least 100 measurements per sample.

Transmission electron microscopy (TEM) observations were carried out with a Jeol JEM 3010 (300 kV) microscope equipped with an LaB_6_ filament (Jeol Ltd., Tokyo, Japan). For analyses, the samples were electrospun on a copper grid and coated with a porous carbon film. All digital micrographs were acquired by an Ultrascan 1000 camera and the images were processed by Gatan digital micrograph (Gatan, Pleasanton, CA, USA).

A Malvern Zetasizer Nano—ZS (Malvern, Worchestershire, UK) was used to measure the zeta potential. All tests were performed using distilled water at room temperature; each sample was measured after 90 s of stabilization at 25 °C, in five replicates.

Thermogravimetric analyses (TGA) were carried out using a TA Instruments Q500 TGA (TA Instruments, New Castle, DE, USA) from 50 to 700 °C under air flow, with a heating rate of 10 °C/min.

### 2.3. Silver Release

The amount of silver released from the electrospun mats over time was monitored via inductively coupled plasma optical emission spectroscopy (ICP-OES) with a PerkinElmer Optima 7000 DV apparatus (PerkinElmer, Waltham, MA, USA). Three specimens per sample of approximately 15 mg were cut from the mats, placed into a centrifugation tube with 10 mL of phosphate-buffered saline (PBS) and then incubated at 37 °C under oscillation for up to 800 h. At different time intervals, the solutions were extracted and digested overnight with nitric acid at a 1:1 ratio by volume. Calibration was conducted using an external standard between 0.5 and 25 ppm.

### 2.4. Antibacterial Test

The PLA/PLA-AgNPs and PLA-PEO/PLA-PEO-AgNPs fibers obtained by electrospinning and morphologically characterized were subsequently assayed to evaluate their antibacterial activity against two reference bacterial strains, purchased from the American Type Culture Collection (ATCC^®^, Manassas, VA, USA). In particular, *Staphylococcus epidermidis* (ATCC^®^ 35984) and *Escherichia coli* (ATCC^®^ 25922), tested as representative Gram-positive and Gram-negative pathogens, were stored in a microbank at −80 °C and cultured at 37 °C on mannitol salt agar (MSA: Oxoid SpA, Rodano, Milan, Italy) and MacConkey agar (MAC: Oxoid SpA) plates, respectively [31].

All polymer nanofiber samples (approx. 15 mg per sample) were sterilized by UV for 20 min, placed in sterile tubes in the presence of a bacterial inoculum of 10^7^ colony-forming-units (CFUs)/mL obtained in Trypticase Soy broth (TSB: Oxoid SpA) and then incubated for different incubation time points (15, 24 and 48 h) at 37 °C. Tubes without fibers, containing only bacteria in broth, were also tested and considered as growth controls. After each incubation time, for all samples, including the growth controls, the number of CFU/mL was quantified by serial plate count into Trypticase Soy agar (TSA: Oxoid SpA) [10].

### 2.5. Statistical Analysis

All the antibacterial tests were performed at least in triplicate, and the results were analyzed by descriptive statistics (mean ± standard error of the mean) and tested by the unpaired *t*-test (Graphpad Prism version 9 for Windows, San Diego, CA, USA). A *p*-value < 0.05 was considered significant.

## 3. Results and Discussion

### 3.1. AgNPs Preparation

The first step of the work was focused on identifying the suitable conditions to obtain AgNPs through a simple procedure that could be integrated with electrospinning. Literature studies [32] indicate dimethyl formamide (DMF) as an adequate reducing agent for silver nitrate, reporting the following reaction mechanism in the presence of water:

HCONMe_2_ + 2Ag^+^ + H_2_O → 2Ag^0^ + Me_2_NCOOH + 2H^+^

The reaction takes place on the carbonyl of the amide group of DMF, which oxidizes to form a carbamate group.

The color of solutions containing silver, or metals in general, depends on the shape and size of the nanoparticles and the surrounding medium; only elements with free electrons possess plasmon resonance in the visible spectrum, thus giving rise to different colors. In the case of nanometric silver (1–50 nm) in DMF, the solution shows an intense yellow color and, as the size of the particles increases, the color changes from yellow, to orange, up to brown/gray, with the increasing particle size.

**Figure 1:**
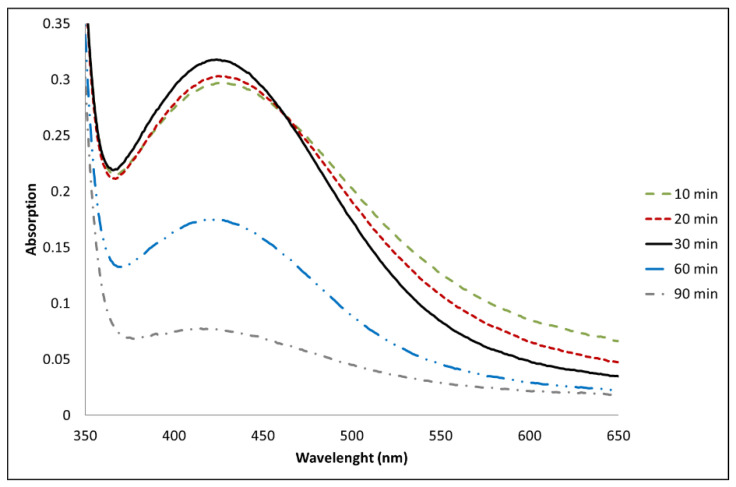
UV-Vis spectrum of a 0.4 M solution of AgNO_3_ in DMF over time: AgNPs in a size range of 10–30 nm give a plasmon resonance peak at 420–430 nm.

In order to fine-tune the times and methods of the reduction process, a 0.4 M solution of AgNO_3_ in DMF at room temperature was prepared and its evolution was followed by UV-Vis spectroscopy, as shown in Figure 1.

By monitoring the absorption at 420 nm, characteristic of Ag nanodispersions, 30 min was identified as the optimal time to achieve an effective reduction under the reaction conditions.

### 3.2. Electrospinning of AgNPs-PLA Fibers

**Figure 2:**
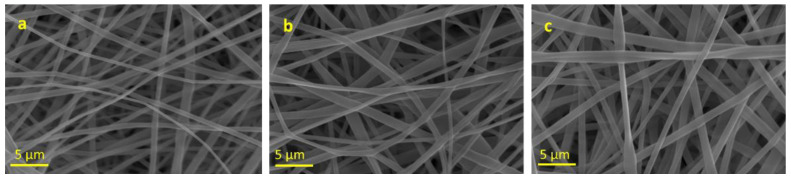
SEM images of PLA fibers in DCM/DMF (9:4 *v*/*v*): (**a**) 7% PLA; (**b**) 7.5% PLA; (**c**) 8% PLA. Average fiber diameters are: (**a**) 0.76 ± 0.13 µm; (**b**) 0.94 ± 0.28 µm; (**c**) 0.95 ± 0.30 µm.

Obtaining good quality fibers from the electrospinning of PLA in DMF is generally challenging [33], mainly due to the limited solubility of PLA in DMF at room temperature and the high boiling point of the solvent. Conversely, DMF exhibits good miscibility with a variety of organic solvents, including chlorinated solvents such as dichloromethane (DCM), in which PLA is well-soluble even at room temperature. The electrospinning of PLA in DCM/DMF blends was then optimized, with the aim of obtaining homogeneous fibers in which to incorporate the AgNPs prepared as described above. Various mixtures of DCM/DMF were prepared, in which PLA was solubilized in different concentrations and the solutions were subjected to electrospinning. The best results were obtained with DCM/DMF mixtures in a volumetric ratio of 9/4 (*v*/*v*) and PLA concentrations in the range 7–8 wt.%. Figure 2 shows SEM images of the electrospun mats obtained from solutions of PLA in DCM/DMF at 7, 7.5 and 8 wt.%, respectively. Smooth, quite homogeneous fibers were obtained in all cases, while the average diameter of the fibers increased slightly with increasing PLA concentration, as it was generally observed due to the increase in entanglement caused by more concentrated solutions [34].

To incorporate the AgNPs, 87.3 mg of AgNO_3_ was dissolved in 4 mL of DMF at room temperature. The solution was left stirring in the dark for 30 min while the color turned to bright yellow. Meanwhile, a solution of 1.184 g of PLA in 9 mL of DCM was prepared. At the end of 30 min, the two solutions were mixed under stirring and immediately subjected to electrospinning. The final composition of the spinning solution was: DCM/DMF 9/4 (*v*/*v*), PLA 7 wt.%, Ag 4.7 wt.% (with respect to polymer).

### 3.3. Characterization of AgNPs-PLA Fibers

**Figure 3:**
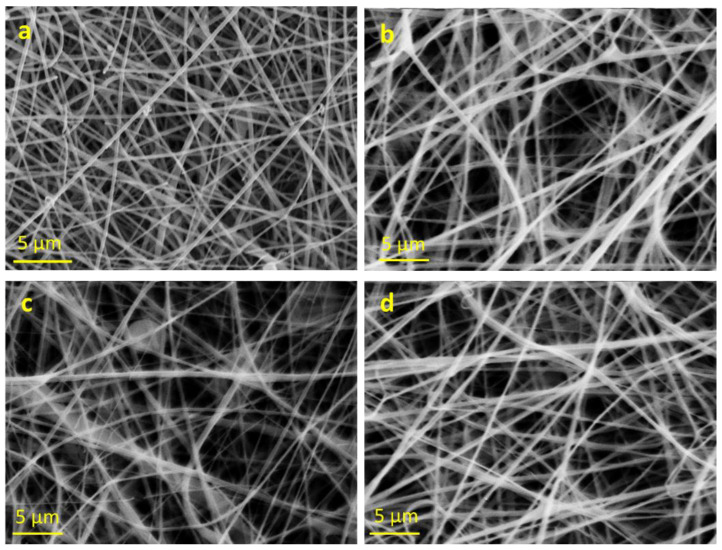
SEM images of PLA—AgNPs fibers, 7% (*w*/*w*) PLA in DCM/DMF: (**a**) Ag/PLA 4.7 wt.%; (**b**) Ag/PLA 3.0 wt.%; (**c**) Ag/PLA 4.0 wt.%; (**d**) Ag/PLA 5.0 wt.%.

The electrospun mat (Figure 3a) showed smooth fibers with a regular morphology and a rather narrow dimensional distribution. The average fiber diameter was 0.46 ± 0.07 µm, significantly smaller than that of pure PLA fibers, as the addition of silver increased the conductivity of the solution, favoring the decrease in the fibers’ diameter [25].

**Figure 4:**
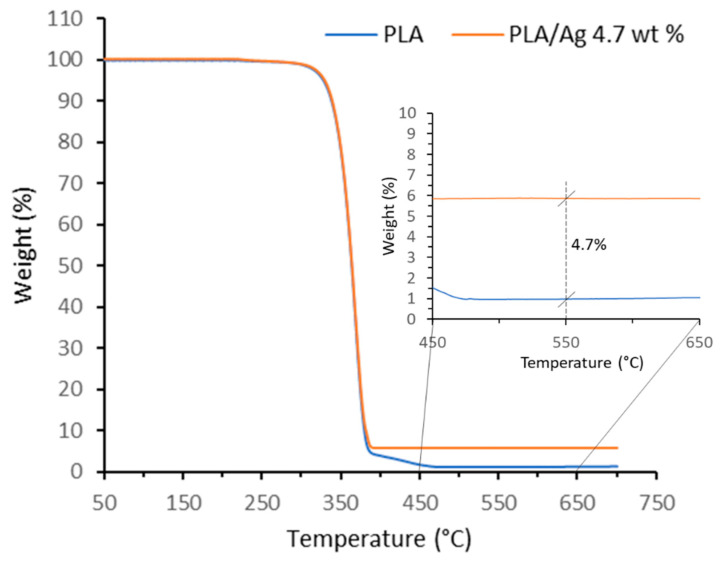
Thermogravimetric degradation curves of PLA and AgNPs/PLA mats.

To double check the amount of silver actually present in the fibers, a portion of the mat was subjected to thermogravimetric analysis in air and the result was compared with the thermogravimetric analysis of pure PLA (Figure 4). The weight difference in the residues at 550 °C (4.7%), after degradation of the PLA matrix, confirms the expected Ag content.

Using the same preparation method and electrospinning conditions and keeping the PLA concentration and the volumetric ratio between the solvents constant, PLA electrospun mats, containing 3.0, 4.0 and 5.0 wt.% Ag, respectively, were prepared (Figure 3b–d). The change in the silver content was not sufficient to produce significant variations in the morphology of the fibers, whose size distribution remained approximately constant around 0.43 ± 0.10 µm.

**Figure 5:**
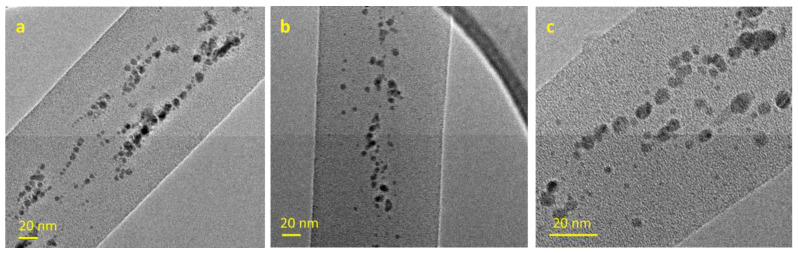
TEM images of PLA—AgNPs fibers, 7% (*w*/*w*) PLA in DCM/DMF, Ag/PLA 5.0 wt.%. (**a**,**b**) low magnification; (**c**) high magnification.

Representative TEM images of PLA/AgNPs nanofibers are presented in Figure 5. The images showed the presence of distinct AgNPs, although aggregates were also visible and a rather large size distribution was found. The average nanoparticle size diameter was 4.96 ± 2.10 nm. It was observed that AgNPs appeared to be more concentrated in the fiber core; this can be explained by considering that the particles were dispersed in DMF, the highest boiling solvent, which was concentrated in the bulk of the fibers, where most of the AgNPs remained.

### 3.4. Silver Release from PLA-AgNPs Fibers

**Figure 6:**
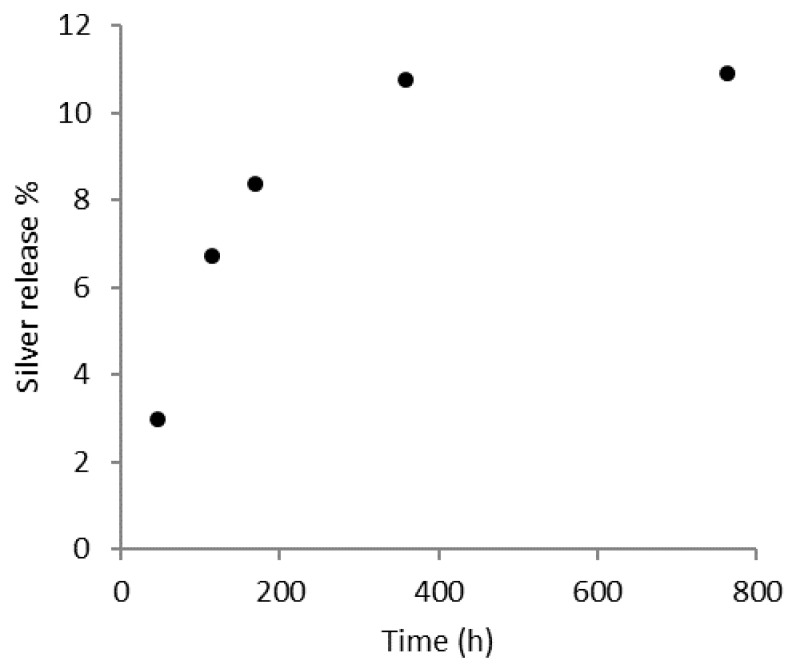
Silver release profile of Ag NPs/PLA (4.0 w%) in PBS at 37 °C (silver released % refers to the total amount of Ag in the fibers).

The release of silver ions over time from PLA-AgNPs (4 wt.%) fibers was monitored by ICP-OES in PBS at pH = 7.2. The trend observed for Ag/PLA 4.0 wt.% is shown in Figure 6, where the percentage of released Ag refers to the total amount present in the fibers. The Ag release grew rapidly in the first 200 h, after which the release rate progressively decreased. The percentage of Ag released after 800 h, compared with the total content in the fibers, was just above 10 wt.%. This behavior was consistent with the TEM observations: AgNPs were concentrated mainly in the core of the fibers and, because the biodegradation of PLA is much slower than the timescale of the experiment, it can be assumed that only the Ag located near the surface of the fibers was rapidly released. Literature studies [35] have shown that the minimum concentration of Ag ions capable of producing any antibacterial effect is of the order of 0.1 mg/L (ppm), while that released in the present experiment varied in the range 1–6 ppm, thus suggesting that the AgNPs/PLA fibers may have some antibacterial effect.

### 3.5. Electrospinning of AgNPs-PLA/PEO Fibers

Although AgNPs/PLA fibers showed sufficient Ag release over time, we wanted to further investigate the possibility of increasing the release in the short term. For this purpose, we explored the possibility of mixing PLA with a water-soluble polymer, whose rapid solubilization in an aqueous environment could ensure a faster release in the short term.

Among water-soluble polymers, PEO is widely used in the biomedical field. Furthermore, PEO was also particularly attractive because literature studies indicate it as a reducing and stabilizing agent for the formation of AgNPs from silver salts [36].

The addition of PEO to the formulation therefore offered the possibility of eliminating the high-boiling DMF from the solvents, as its role as a reducing agent was no longer necessary. However, although PEO is well-soluble in DCM, this is not true for silver nitrate, and the first tests, carried out with DCM as the only solvent, gave poor results in terms of Ag concentration in the fibers. The formulation was then integrated with acetonitrile (ACN), which can adequately solubilize both PEO and AgNO_3_ and whose boiling point is considerably lower than that of DMF. In total, 51.5 mg of PEO was completely solubilized in 2 mL of ACN under stirring. Next, 32.4 mg of AgNO_3_ was added and the solution was monitored with UV-Vis until the complete reduction of Ag^+^, after about 60 min. Meanwhile, 0.463 g of PLA was solubilized in 4 mL of DCM. Then, the two solutions were mixed under stirring and immediately subjected to electrospinning. The final composition of the spinning solution was: DCM/ACN 2/1 (*v*/*v*), PLA/PEO (9/1, wt./wt.) 7 wt.%, Ag 4.0 wt.% (with respect to polymers).

However, the electrospinning of this solution immediately presented dripping problems, resulting in poor efficiency. In fact, the solvent change and the addition of PEO resulted in a visible decrease in the viscosity of the solution. The concentration of the polymers in solution was therefore increased and further tests were carried out, keeping the Ag content constant (4.0 wt.%, with respect to polymers), with a concentration of PLA/PEO of 7.5 wt.% and 8.5 wt.%, respectively. A further attempt was made by decreasing the flow rate of the solution and a third experiment was conducted with a PLA/PEO concentration of 7.5 wt.% and a flow rate of 0.3 mL/h.

### 3.6. Characterization of AgNPs-PLA/PEO Fibers

**Figure 7:**
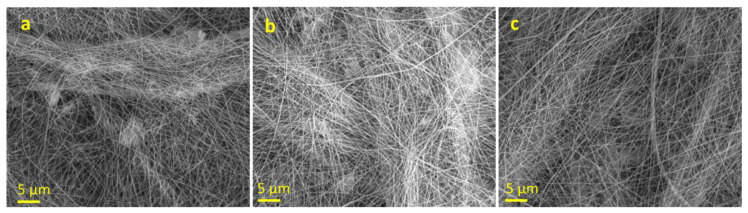
SEM images of PLA/PEO (9:1)—AgNPs fibers (4.0 wt.% Ag): (**a**) PLA/PEO 7.5 wt.%, 0.5 mL/h; (**b**) PLA/PEO 7.5 wt.%, 0.3 mL/h; (**c**) PLA/PEO 8.5 wt.%, 0.5 mL/h. Average fiber diameters are: (**a**) 0.21 ± 0.06 µm; (**b**) 0.18 ± 0.04 µm; (**c**) 0.15 ± 0.06 µm.

Both solutions at 7.5 wt.% (Figure 7a,b) still presented dripping problems during spinning, although they returned a fairly good deposition of fibers of rather homogeneous diameter. The solution at 8.5 wt.% (Figure 7c), on the other hand, proved to be adequate and resulted in a good deposition of fibers with homogeneous dimensions. Moreover, it was immediately evident that by using acetonitrile as a solvent instead of DMF, fibers with much smaller average diameters were obtained; this was considered an advantage in terms of the surface/volume ratio of the fibers for the silver release.

**Figure 8:**
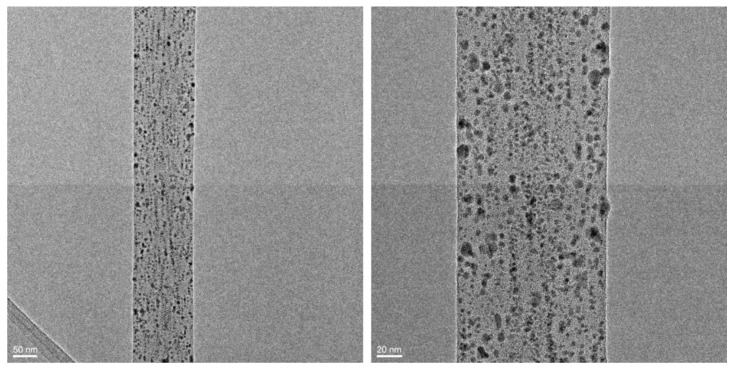
TEM images of PLA/PEO (9:1)—AgNPs fibers (4.0 w% Ag).

Figure 8 shows representative TEM micrographs of the sample in Figure 7c. It is apparent that the dispersion of AgNPs in the fibers produced using PLA/PEO in ACN/DCM was much more homogeneous than that of samples obtained from PLA alone in DMF/DCM. In particular, the mean diameter of the AgNPs was 3.95 ± 1.00 nm.

### 3.7. Silver Release from PLA/PEO-AgNPs Fibers

**Figure 9:**
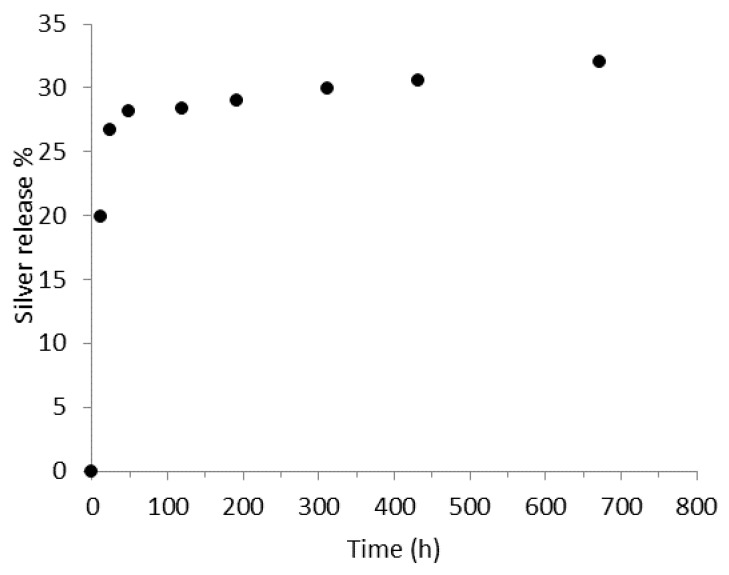
Silver release profile of PLA/PEO AgNPs (4.0 wt.%) in 25 mM PBS (pH = 7.2) at 37 °C.

Again, the release of Ag ions over time from PLA/PEO-AgNPs fibers was monitored by ICP-OES in PBS (pH = 7.2). The observed trend is shown in Figure 9, where the % released Ag refers to the total amount present in the fibers.

The Ag release appeared to be very rapid in the first 24–48 h, reaching just under 30% of the total quantity of Ag present in the fibers. Thereafter, the release rate drastically decreased, settling at around 33% after more than 700 h of testing. This behavior can be attributed to the presence of PEO which, being water soluble, dissolves in the first hours of testing, facilitating the Ag release. Thereafter, the Ag dissolution is made possible by the slow PLA degradation.

### 3.8. Antibacterial Effect

The antibacterial effect of the nanofibers prepared from PLA, PLA/PEO, PLA-AgNPs and PLA/PEO-AgNPs was evaluated against both Gram-positive and Gram-negative bacteria by using CFU/mL counting during different incubation time points, specifically 15, 24 and 48 h.

Our results demonstrated that no antibacterial activity was detected for both pure PLA and pure PLA/PEO nanofibers used as control materials, since the CFU/mL overlapped those of bacteria in the absence of any type of nanofibers (growth controls). These results, in line with those of literature papers [37,38], revealed no intrinsic antimicrobial action of PLA-based electrospun nanofibers against different pathogens.

In contrast, for the AgNPs-loaded PLA and PLA/PEO nanofibers, a significant (*p* < 0.05 or *p* < 0.001) growth inhibitory effect against both *S. epidermidis* and *E. coli*—highlighted by a reduction in the CFU/mL counts—was observed during the course of the experiments, confirming the antibacterial effect of Ag released from the samples.

**Figure 10:**
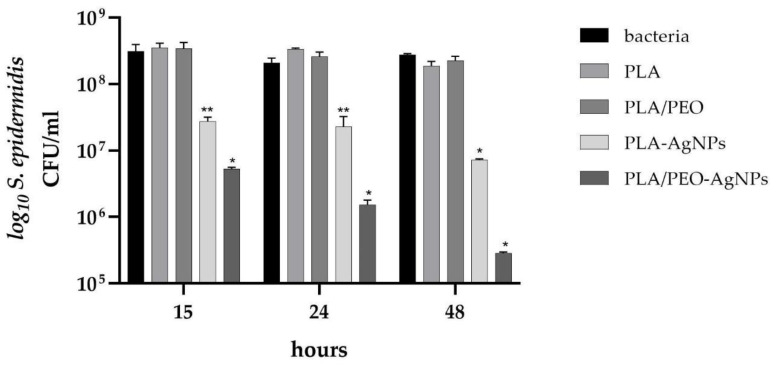
Number of *S. epidermidis* (log_10_ colony forming unit, CFU/mL) in presence of PLA and PLA/PEO nanofibers loaded with silver nanoparticles (AgNPs) after 15, 24 and 48 h of incubation. Results are the mean values ± standard error of the mean (SEM) of six independent experiments. ** *p* < 0.05 or * *p* < 0.001 vs. bacteria, PLA or PLA-PEO, unpaired *t*-test.

As reported in Figure 10, a reduction in staphylococcal growth starting from 15 h up to 48 h of incubation for both PLA and PLA/PEO loaded with AgNPs was detected, with a stronger effect exerted by the latter, highlighted by a decrease in the CFU/mL values overtime. In fact, at 15 and 24 h, in the presence of pure PLA and pure PLA/PEO CFU/mL staphylococci reached CFU values of about 3.40 × 10^8^ and 3.00 × 10^8^, respectively, whereas a significant decrease to about 5.00 × 10^7^ CFU/mL for PLA-AgNPs (*p* < 0.05) and to about 3.40 × 10^6^ CFU/mL for PLA/PEO-AgNPs (*p* < 0.001) was achieved. A further significant (*p* < 0.001) reduction was observed at 48 h of incubation, highlighted by *S. epidermidis* counts of about 2.00 × 10^8^ CFU/mL for the controls of pure nanofibers, and 7.20 × 10^6^ and 2.80 × 10^5^ CFU/mL for PLA-AgNPs and PLA/PEO-AgNPs, respectively.

**Figure 11:**
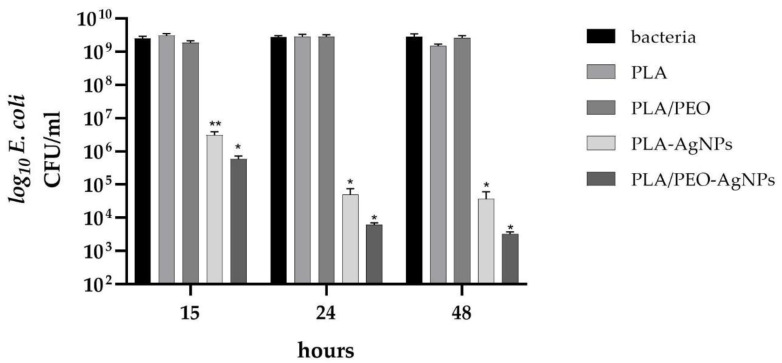
Number of *E. coli* (log_10_ colony forming unit, CFU/mL) in presence of PLA and PLA/PEO nanofibers loaded with silver nanoparticles (AgNPs), after 15, 24 and 48 h of incubation. Results are the mean values ± standard error of the mean (SEM) of six independent experiments. ** *p <* 0.05 or * *p* < 0.001 vs. bacteria, PLA or PLA-PEO, unpaired *t*-test.

A similar antibacterial trend was obtained by assaying the Gram-negative bacteria (Figure 11). In the presence of Ag, *E. coli* significantly (*p* < 0.05 or *p <* 0.001) decreased its growth, reaching CFU/mL values of 3.07 × 10^6^, 5.00 × 10^4^ and 3.66 × 10^4^ in the presence of PLA-AgNPs and of 5.91 × 10^5^, 6.27 × 10^3^ and 3.28 × 10^3^ in the presence of PLA/PEO-AgNPs after 15, 24 and 48 h, respectively, when compared with about 2.44 × 10^9^ CFU/mL of the pure nanofibers.

Altogether, the excellent results obtained by assaying the antibacterial effect of the AgNPs-loaded nanofibers, are well in agreement with those of the available scientific literature [23,24,37,39,40,41]. Mahapatra A. et al. (2012) prepared polyacrylonitrile/Ag nanofibers, Shi Q. et al. (2011) synthetized nylon 6/Ag nanofibers and both revealed an antibacterial action of the enriched samples [23,24]. In the research of Chae et al. (2011), an antibacterial effect of AgNPs-loaded nanofibers made of β-cyclodextrin and polyacrylonitrile was demonstrated, and a more pronounced antibacterial activity, as optical density, was achieved against *E. coli*, with respect to *S. epidermidis* [40]. These data are similar to those obtained in the present work; however, we were also able to quantify the growth reduction as CFU/mL count. Several literature reports regarding the controlled silver ion release including the use of hydrogels, synthetic polymers, nanofibers, nanotubes, mesoporous layers, celluloses and cap layers are available [11,12,31,42,43,44,45,46]; however, most of them, using the disk diffusion test or even a CFU count, are difficult to compare with the data reported here. Despite this methodological issue, different authors demonstrated a pronounced antibacterial activity of these nanodevices enriched with silver against various microorganisms [43,44,45,46]. In particular, to delve into mats comparable to those produced in this paper, in a recent study, Wei W. et al. [45] obtained polyvinyl alcohol electrospun nanofibers doped with monodisperse AgNPs, via the one-pot reactions, and revealed a good antibacterial efficacy against *S. aureus* by the inhibition zone assay. Similarly, Li J et al. [46] investigated the deposition of AgNPs on dialdehyde nanofibrillated cellulose, prepared by the oxidation of nanofibers, and highlighted an efficient antibacterial activity against both *S. aureus* and *E. coli*, by disc diffusion method.

The use of silver as an antibacterial agent has received a great deal of attention over the past twenty years. However, despite the many hypotheses available, there is still no consensus on the main mechanism involved in the antibacterial properties of AgNPs [30,35,47]. The eradication of bacteria may result from either silver nanoparticles themselves, released silver ions or a synergistic effect of both [47]. Based on the large amount of available literature, the proposed mechanisms involve: (I) adhesion of AgNPs onto the bacterial membrane, with modification of the lipid bilayer and increase in the membrane permeability; (II) intracellular penetration of AgNPs; (III) induced cellular toxicity triggered by the generation of reactive oxygen species (ROS) and free radicals and (IV) modulation of intracellular signal transduction pathways towards apoptosis [30]. A number of critical parameters, such as ion release ability, surface area, surface charge, concentration, etc., can influence the antibacterial properties of AgNPs. The release rate of silver ions depends on many factors, including the size and shape of NPs and their colloidal state. In particular, small-sized, irregularly shaped AgNPs with large surface areas generally show more toxicity and exhibit a faster ion release rate [30]. The adverse effect of silver ions is due to interactions with thiol and amino groups of proteins with nucleic acids and with cell membranes [35]. Additionally, released silver ions bind to and alter several enzymes, which are crucial for cellular respiration and metabolism, promote ROS generation and oxidative stress and interfere with DNA through cell division and replication [10,39,47,48,49,50]. Overall, given the high number of variables involved, all possible concurrent mechanisms and the experimental conditions in vitro, different from those (more complex) of biological environments, many authors agree that it is generally not possible to identify a single, predominant cause for the antimicrobial action of AgNPs [30,35,40,47,51].

The designed, prepared and characterized PLA-AgNPs or PLA/PEO-AgNPs nanofibers obtained in the present study were capable of releasing a silver amount able to counteract in a more efficient manner the *E. coli* growth with respect to *S. epidermidis* one. These data reflect the Ag’s ability to act directly on both types of bacteria, even if, due to their differences on the external layers, this action is more pronounced on Gram-negative ones [39,52]. In addition, the AgNPs synthesized in our study had a size < 10 nm, and it was found that small-sized AgNPs showed a higher release rate of silver ions, particularly in Gram-negative bacteria [30]. Several literature studies have also shown that surface charge plays an important role in the interaction with bacteria [30,53,54]. Chwalibog et al. [55] demonstrated that nanoparticles with negative zeta potential were particularly efficient, being able to disintegrate the cell walls and cytoplasmic membranes and releasing cytoplasm outside the cell. Our AgNP-containing mats exhibited a negative zeta potential of −19.1 ± 0.8 mV, and this certainly contributed to their antimicrobial activity. The hypothesis that the addition of PEO as a water-soluble polymer allowed the increase of the antimicrobial effect in the short term was assayed; in fact, we demonstrated an enhanced antibacterial activity of the PLA/PEO-AgNPs nanofibers, compared with PLA-AgNPs ones, thanks to a more efficient short-term Ag release from the former.

Since we mainly focused on developing a simple method to prepare biodegradable mats with antibacterial effect, one limitation of our study is the lack of information about the cytotoxicity of the products. As reported by several authors [10,39], the toxic action of AgNPs towards bacteria and eukaryotic cells occurs in the same concentration range, making the usability window rather tight. On the other hand, some authors question the validity of in vivo cytotoxicity tests which, in the absence of a complex physiological environment and the body’s immune response, cannot be considered as the ultimate predictors of in vivo conditions [28]. Furthermore, it has also been demonstrated that the cytotoxicity of silver-containing materials can be exploited to simultaneously obtain antimicrobial and anti-proliferative actions in malignant cell lines, such as those of skin cancer [29].

Our aims were to identify an easier method for the preparation of antimicrobial mats than those already present in the literature and to demonstrate that variations in the mixture of polymers used could ensure a tunable release of Ag that could be adapted to different needs. Interestingly, in several literature studies [25,56], mats releasing similar amounts of Ag to those in our study performed very well in cytotoxicity tests, showing no adverse effects. This indicates that the products of this study show good potential for applications as biomaterials. Further studies designed for specific applications such as those in wound dressing, will be needed to investigate this aspect in detail.

## 4. Conclusions

In the present research, we successfully designed and prepared PLA and PLA/PEO nanofibers loaded with silver nanoparticles. We have developed a one-step preparation method that allows the obtaining of AgNPs by the reduction of AgNO_3_, simultaneously with the preparation of the solution for electrospinning, thus avoiding the complex and time-consuming steps of post-processing reduction. The SEM and TEM characterization of the nanofibers has shown that, by optimizing the composition of the precursor solutions, it is possible to obtain homogeneous fibers containing AgNPs and that the introduction of PEO in the blend allows one to obtain both a narrower dimensional distribution and a better spatial distribution of the NPs in nanofibers. The silver release from nanofibers over time was found to be influenced by their composition, with the nanofibers containing a water-soluble polymer (PEO) fraction ensuring the release of up to just under 30% of their silver content in 48 h. Accordingly, the microbiological results revealed a strongest growth inhibitory effect in the short term of the PLA/PEO-AgNPs, with respect to PLA-AgNPs, against both *S. epidermidis* and *E. coli*, with a more pronounced action towards the Gram-negative bacteria, since the thicker peptidoglycan layer around the Gram-positive bacteria seem to partially protect these microorganisms from the silver activity.

Overall, the nanofibers produced here showed excellent antibacterial efficacy; moreover, the possibility of modifying the system by adjusting the ratio between PLA and PEO potentially allows the regulation of the silver release according to the requirements of the most diverse biomedical applications.

## Data Availability

The authors confirm that the data supporting the findings of this study are available within the article and/or on request from the corresponding author (P.B.).
